# New investigations around *CYP11A1 *and its possible involvement in an androstenone QTL characterised in Large White pigs

**DOI:** 10.1186/1297-9686-43-15

**Published:** 2011-04-19

**Authors:** Annie Robic, Guillaume Le Mignon, Katia Fève, Catherine Larzul, Juliette Riquet

**Affiliations:** 1 INRA, UMR444, Laboratoire de Génétique Cellulaire, 31326 Castanet-Tolosan, France; 2 INRA, UMR1313, Génétique Animale et Biologie Intégrative (GABI), 78352 Jouy-en-Josas, France

## Abstract

**Background:**

Previously, in boars with extreme androstenone levels, differential expression of the *CYP11A1 *gene in the testes has been characterised. *CYP11A1 *is located in a region where a QTL influencing boar fat androstenone levels has been detected in a Large White pig population. Clarifying the role of CYP11A1 in boar taint is important because it catalyses the initial step of androstenone synthesis and also of steroid synthesis.

**Results:**

A genome-wide association study located *CYP11A1 *at approximately 1300 kb upstream from SNP H3GA0021967, defining the centre of the region containing the QTL for androstenone variation. In this study, we partially sequenced the *CYP11A1 *gene and identified several new single nucleotide polymorphisms (SNP) within it. Characterisation of one animal, heterozygous for *CYP11A1 *testicular expression but homozygous for a haplotype of a large region containing *CYP11A1*, revealed that variation of *CYP11A1 *expression is probably regulated by a mutation located downstream from the SNP H3GA0021967. We analysed *CYP11A1 *expression in LW families according to haplotypes of the QTL region's centre. Effects of haplotypes on *CYP11A1 *expression and on androstenone accumulation were not concordant.

**Conclusion:**

This study shows that testicular expression of *CYP11A1 *is not solely responsible for the QTL influencing boar fat androstenone levels. As a conclusion, we propose to refute the hypothesis that a single mutation located near the centre of the QTL region could control androstenone accumulation in fat by regulating the *CYP11A1 *expression.

## Background

Boar taint refers to an unpleasant odour and flavour of meat which occurs in a high proportion of uncastrated male pigs and is primarily due to the accumulation of androstenone and skatole in fat tissue [1,2]. Androstenone is synthesised in the testis, together with the steroid hormones, androgens and estrogens, from pregnenolone [3-5], in relation to sexual development and is stored in fat tissue because of its lipophilic properties.

Currently, only a few studies have tried to identify QTL for androstenone accumulation [6-9]. It is important to understand the genetic mechanisms controlling this trait in order to be able to select pigs for low androstenone levels and thus limit the occurrence of boar taint.

Le Mignon et al. [10] identified QTL for androstenone variation in a 480 Large White (LW) pig population using the Illumina PorcineSNP60 BeadChip. The present study focused on one of these QTL, explaining 18.7% of the genetic variance, which was detected on the q-arm of chromosome *Sus scrofa *7 (SSC7) using GWAS (Genome Wide Association Studies) near the position 66 Mb on the "*Sscrofa9*" version (April 2009) of the pig genome sequence. Examination of the gene content in this QTL region, suggested *CYP11A1 *as an obvious candidate gene. Moe et al. [11] and Grindfleck et al. [12] had already detected differential expression of *CYP11A1 *in the testes of boars with either extremely high and or low levels of androstenone in fat. Moreover, a previous study reported one polymorphism in exon 1 of *CYP11A1 *significantly associated with androstenone levels in Yorkshire boars [13]. This gene encodes the CYP11A1 enzyme, which is localized in the mitochondrial inner membrane, and catalyses the conversion of cholesterol to pregnenolone in the first and rate-limiting step of the synthesis of steroid hormones [14]. Therefore, it is very important to clarify the role of *CYP11A1 *in boar taint. If testicular expression of *CYP11A1 *is found to influence the QTL for androstenone variation, it would be difficult to select against this QTL without encountering reproduction problems.

## Methods

### Animals and samples

On the INRA experimental farm, 98 LW sows were inseminated with 56 LW boars, chosen as unrelated as possible. Each boar inseminated one or two sows. A total of 580 male piglets were raised in pens till they reached 110 kg of live body weight and then slaughtered in a commercial slaughterhouse. A total of 480 animals were measured for backfat androstenone levels.

Six litters were produced by inseminating LW sows with semen from two commercial LW boars. Twenty-two animals were produced and then slaughtered at 24-25 weeks of age. Testicular samples were collected immediately after slaughter, frozen in liquid nitrogen and stored at -80°C. To obtain testicular samples, testes were decapsulated to remove connective tissues, fasciae and the main blood vessels. Samples (2 to 5 cm^3^) were collected from the inner part of the testicular tissue, containing Leydig cells.

### Real time PCR

Samples were disrupted, homogenised and ground to a fine powder by rapid agitation for 1 min in a liquid-nitrogen-cooled grinder with stainless steel beads before RNA extraction. Total RNA was isolated from testis using *Total Quick RNA *(Talent) kits according to the manufacturers' instructions, and treated with DNase to remove contaminating DNA. RNA concentration was determined using the NanoDrop ND-1000 spectrophotometer (NanoDrop Technologies, DE, USA). First strand cDNA synthesis was conducted using SuperScript™-II Rnase H- Reverse Transcriptase (Invitrogen, Carlsbad, CA). According to the manufacturer's instructions, 0.5 μg of total RNA from each sample was used as a template with dN9 random primers (Ozyme, New England Biolabs), in a total volume of 100 μL.

The level of *CYP11A1*expression was determined by real-time PCR on cDNA from testes. Experiments were performed on the ABI 7900HT (Sequence Detection System 7900HT) in a 384-well plate. All measurements were performed in duplicate on the same plate and no reference sample was used. Primers were designed in exon 3 (TGTTTCGCTTCGCCTTTGA) and exon 4 (CCCAGGCGCTCTCCAAAT) of *CYP11A1 *cDNA. Transcript's concentrations were corrected with respect to the housekeeping gene, *TOB2B *(GGGATGTCTGAAGAAGTACGAAAC//CATTCCTACAAGCCATTCCTTACG). Data was analysed with ABI software to obtain Ct values (threshold cycle). Four points of dilutions of a mix of cDNA were used for each gene and for each tissue, to determine PCR efficiency (E). As efficiency levels were similar for all measured genes (including the reference gene), results are expressed as E^(Ct_ref - Ct_gene) ^× 1000 in arbitrary units.

Before quantifying *CYP11A1 *transcripts, possible alternative transcripts were compiled from databases and we checked that the alternative transcript (AK235955 or DB787788) with a 3' shortcut exon 3 was absent in the testicular cDNA.

### Sequences

Sequencing of BAC CH242-402H17 containing *CYP11A1 *gene related sequences is underway and sub-clone sequences are being captured by a blast procedure (http://www.ncbi.nlm.nih.gov/genome/seq/BlastGen/BlastGen.cgi?taxid=9823) from the "traces-other" database.

To find polymorphisms, PCR products were produced with genomic DNA from several animals. To sequence the PCR products, an aliquot (1 to 12 μL) was purified by a single treatment (45 min at 37°C followed by 30 min at 80°C) using 0.5 U of Shrimp alkaline phosphatase (Promega) and 0.8 U of exonuclease I (New England Biolabs). Sequencing was done with a 3730 ABI capillary DNA sequencer using a Big Dye terminator V3.1 cycle sequencing kit.

### Statistical analysis

Differences between two groups of animals were assessed with a heteroscedastic Student's t-test as proposed by MS-Excel (Microsoft Corporation).

## Results and discussion

### QTL region

**Figure 1:**
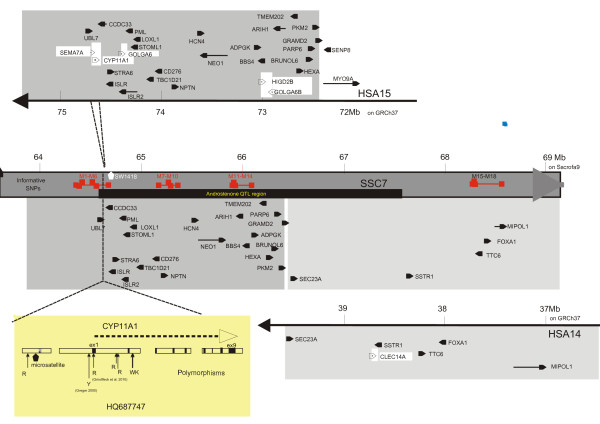
**Schematic representation of the SSC7 and human homologous regions**. Center part: schematic representation of genes in the 64-69 Mb SSC7 region (in accordance with the *sscrofa9 *draft sequence); top left hand side: HSA15 segment homologous to the left half of this SSC7 region; lower right hand side: HSA14 segment homologous to the right half of this SSC7region; each gene sequence is represented by an arrow i.e. full arrow if homologous porcine gene exists and hollow arrow if not; lower left hand side: representation of part of the porcine sequence gap including *CYP11A1 *with its structure schematized showing the location of the polymorphisms characterised in this study i.e. one SNP (R in IUPAC codification) and one microsatellite identified in the distant 5' flanking sequence; SNP (Y) patented by Greger [18] was found in the proximal promoter and SNP (R) previously characterised by Grindfleck et al. [12] was found in the first exon; two new SNP (R and R) in the first intron and two consecutive SNP (WK) in the second intron were also detected; composition and position in Mb (*Sscrofa9*) of SNP marker groups: (1) over the 64.38-64.65 Mb region with M1 = ALGA0042289; M2 = INRA0026201; M3 = ASGA0034277; M4 = DRGA007689; M5 = ALGA0042294; M6 = H3GA0021937; (2) over the 65.13-65.33 Mb region with M7 = ASGA0034288; M8 = INRA0026223; M9 = ALGA0042315; M10 = ASGA0034291; (3) over the 65.91-66.11 Mb region with M11 = H3GA0021967; M12 = ASGA0034309; M13 = ASGA0034310; M14 = MARC0076146 and (4) over the 68.27-68.56 Mb region with M15 = INRA0026286; M16 = MARC0099388; M17 = inra0026290; M18 = ALGA0042359

The 18 informative SNP present in the region containing the QTL for androstenone variation were classified into four groups based on their positions in Mb (Figure 1). The position of this QTL was arbitrarily defined in a window of 3 Mb around the most highly associated marker, H3GA0021967 (named M11 and located at 65.91 Mb on the *"Sscrofa9*" provisional genome sequence), identified by GWAS analysis [10]. Since sequencing of the porcine genome is not yet completed [15], our results are anchored on the human map. The genomic content of this region (64.4 - 67.4 Mb) was deduced from the global alignment proposed by the Narcisse software [16]. The first part of the QTL region between 64.40 and 66.45 Mb corresponded to the *CYP11A1*-*GRAMD2 *region from HSA15 (Figure 1) and the second part between 66.45 and 67.40 Mb was homologous to HSA14 (*SEC23A-SSTR1*). The information was completed and compared to the provisional annotation of the "*Sscrofa9"*version available on the Ensembl web site (http://www.ensembl.org/). We found that the assembly between 66.0 and 66.4 Mb did not coincide completely although the SNP order was correct (Figure 1). Moreover, four small gaps were detected (Figure 1) and in particular, close to the porcine position 64.6 Mb. Using the Blast procedure available on the Ensembl web site, we showed that the 55 kb fragment on "*Sscrofa9" *separating the extremities of *UBL7 *and *CCDC33 *genes did not contain sequences related to the *SEMA7A *and *CYP11A1 *genes. Curiously, on the human sequence, this region extended over a total of about 73 kb and contained these two genes which did not overlap but extended over 25 and 30 kb, respectively.

### Porcine *CYP11A1 *gene

*CYP11A1 *appeared to be a promising candidate gene for the QTL for androstenone variation since its product, the CYP11A1 enzyme, catalyses the initial step of androstenone synthesis. Moreover, Moe et al. [11] and Grindfleck et al. [12] had already detected differential expression of *CYP11A1 *in the testes of boars with various extreme androstenone levels in fat. The cDNA of *CYP11A1 *is known in pig (NM_214427) and the sequencing of BAC CH242-402H17 is underway. We performed several assemblies of sub-clone sequences starting with exon sequences. To capture the 5' flanking sequence, we used human sequences and in particular a regulatory region (HSA15:74665162-74667983). We were able to propose an initial assembly (HQ687747) with four genomic fragments schematized on Figure 1. *CYP11A1 *gene is composed of nine exons in most mammals. Pig intron 1 (3205 bp) is significantly shorter than the human counterpart (19261 bp) but longer than the mouse one (975 bp). To characterise polymorphisms, the corresponding human 19 kb long intron 1 was sequenced in several animals chosen from the 480 LW animals. One SNP (R according to IUPAC codification) and one microsatellite were identified in the distant 5' flanking sequence. SNP (Y) patented by Greger [17] was found in the proximal promoter and SNP (R), previously characterised by Grindfleck et al. [12], was found in the first exon. In the first intron, two new SNP (R and R) and in the second intron, two consecutive SNPs (WK) were detected. Thus, seven SNP and two microsatellites (with SW1418) were available to explore the *CYP11A1 *region.

### Expression of *CYP11A1*

It would have been interesting to analyse variations of the testicular *CYP11A1 *expression in animals from this LW population but no samples were available. Fortunately, expression of *CYP11A1 *in the testis of animals from other LW families could be estimated by real-time PCR. The results are shown in Table 1. In family A, the level of *CYP11A1 *expression ranged between 200 and 600 A.U. in the seven animals analysed, while in family B, two animals with a very high level of *CYP11A1 *expression (1425) and two animals with a low level (250) were found. Moreover four microsatellites were genotyped around *CYP11A1 *[see additional file 1, Table S1], which allowed to deduce that the boar is homozygous -/-, the sow (61043) of family A is homozygous -/- and the sow (65472) of family B is heterozygous +/- for *CYP11A1*expression level. Nevertheless, it is very likely that this latter sow (65472) is homozygous for the entire haplotype over this region [see additional file 1, Table S1].

**Table 1:** Testis *CYP11A1 *expression in two LW families

		qPCR on RT products of RNA from testis			[CYP11A1]
	Haplotypes SSC7/SSC7		Descendant	Haplotypes SSC7/SSC7	qPCR Arbitrary units
					
Boar 1 Pat1/Pat2	Family A	Dam 61043	75082	Mat1/Pat2	463
Boar 1 Pat1/Pat2	Family A	Mat1/Mat2	75083	Pat2/Mat1	224
Boar 1 Pat1/Pat2	Family A		75084	Pat1/Mat2	586
Boar 1 Pat1/Pat2	Family A		75085	Pat1/Mat2	199
Boar 1 Pat1/Pat2	Family A		75086	Pat1/Mat1	260
Boar 1 Pat1/Pat2	Family A		75087	Pat2/Mat2-Mat1	604
Boar 1 Pat1/Pat2	Family A		75088	Pat2/Mat2	408
					
Boar 1 Pat1/Pat2	Family B	Dam 65472	75010	Pat1/Mat3	1440
Boar 1 Pat1/Pat2	Family B	Mat1/Mat3	75011	Pat2/Mat3	1410
Boar 1 Pat1/Pat2	Family B		75012	Pat2/Mat1	273
Boar 1 Pat1/Pat2	Family B		75014	Pat2/Mat1	227

In this table we report the results of the quantification of *CYP11A1 *expression in the testis [Cyp11A1]; contrary to animals in family A, in family B, the level of *CYP11A1*expression in descendants could be distinguished as a function of their maternal SSC7 chromosome; for more details on the characterisation of SSC7, [see additional file 1, Table S1] and for the quantification of *CYP11A1 *expression on testes for all the animals of the six families see Table 3

### Haplotype analysis in the CYP11A1 region

Sow 65472 was genotyped for the 21 SNP (M1-M14 and 7 SNP related to *CYP11A1*) and for two microsatellites (located 5' of *CYP11A1 *and SW1418). With the exception of M12, M13 and M14, all the markers were homozygous (data not shown). This female appeared to be homozygous for a large region (M1-M11) including SNP H3GA0021967. We examined the haplotypes of 480 LW animals for 14 SNP (M1 to M14) from the *CYP11A1 *region and found that 14 animals shared the same two SSC7 chromosomal regions present in sow 65472. Nevertheless, genotyping for three markers inside the *CYP11A1 *gene did not detect any animal carrying the 65472 sow's haplotype. Thus, we believe that the number of genotyped SNP is sufficient to assume that sow 65472 is homozygous for a large region (M1-M11) including SNP H3GA0021967.

Since sow 65472 is considered as heterozygous for *CYP11A1 *expression level and homozygous for a haplotype in the M1-M11 large region, we hypothesized that the mutation controlling *CYP11A1 *expression is located downstream M11. This region was superimposed on the androstenone QTL region, which enabled us to suggest that a unique mutation located near the centre of the QTL region (M11-M14) could control androstenone accumulation in fat by regulating the *CYP11A1 *expression.

### Haplotype analysis in the centre of the QTL region

We examined the haplotypes between M11-M14 in 480 LW animals (Table 2). The GGAG haplotype in the third group of SNP (M11-M12-M13-M14) occurred at a high frequency in the population (0.48) and had a negative effect on androstenone level [10]. Since homozygous GGAG animals had a statistically significant lower level of androstenone than animals GGAG/TAGA or TAGG/TAGG (Table 2), we suggest that haplotypes TAGA and TAGG could have a positive effect on androstenone level.

**Table 2:** Effects of various haplotypes of the M11-M14 region on Androstenone accumulation (480 LW)

**1**^**rst **^**group of animals with haplotypes**				**2**^**nd **^**group of animals with haplotypes**			
			
nb animals	Haplotype SSC7	[andro]	*P*	nb animals	Haplotype SSC7	[andro]	Effect on [andro]
303	GGAG/X or GGAG/GGAG	-0.11 +/- 0.63	4.09E-07	116	X/X	0.26 +/- 0.67	GGAG = andro -
							
102	GGAG/GGAG	-0.25 +/- 0.55	0.00237	75	GGAG/TAGA	0.04 +/- 0.68	TAGA = andro +
							
102	GGAG/GGAG	-0.25 +/- 0.55	0.000822	22	TAGG/TAGG	0.36 +/- 0.71	TAGG = andro +

Analysis of the M11, M12, M13 and M14 SNP set revealed five haplotypes (TAGA, TAGG, TGAG, GGAA and X for haplotypes other than these four) in the 480 animals of the LW population used for QTL mapping; [andro] is the mean +/- SD of androstenone level in fat after transformation in log; *P *= Student's *t*-test

Furthermore, we analysed *CYP11A1 *expression in LW families according to haplotypes specifically of the QTL region. Haplotypes of the region between markers M11 and M14 in 22 animals characterised for *CYP11A1 *expression are shown in Table 3. Only two animals (75010 and 75011) considered as heterologous for *CYP11A1 *expression level had haplotype TGAG, the fourth haplotype characterised in the 480LW population and for which it was not possible to evaluate its effect on androstenone accumulation. Nevertheless these two animals have a paternal haplotype GGAG or TAGG which could have a contrary effect on androstenone level. Moreover, we found six animals GGAG/TAGA expected as +/- and seven animals TAGG/TAGA expected as +/+ for androstenone accumulation with no significant difference in *CYP11A1 *expression (Table 3). Haplotypes of the M11-M14 region in these 22 LW animals characterised for *CYP11A1 *expression level are not concordant with those of the 480 LW animals. Effects of the QTL region's haplotype on *CYP11A1 *expression level and androstenone accumulation are different.

**Table 3:** Haplotypes of the M11-M14 region in 22 LW animals from six families

					Androstenone QTL			[CYP11A1]	
					
					haplotypes			real time PCR	
							
Boar	Sire haplotype	family	Sow	Animal	Paternal allele	Maternal allele	Expected (andro)	Individual value	Mean
boar 1	TAGG/GGAG	A	61043	75082	TAGG	TAGA	+/+	463	361 +/- 463
boar 1	TAGG/GGAG	A	61043	75083	TAGG	TAGA	+/+	224	361 +/- 463
boar 1	TAGG/GGAG	A	61043	75087	TAGG	TAGA	+/+	605	361 +/- 463
boar 1	TAGG/GGAG	A	61043	75088	TAGG	TAGA	+/+	408	361 +/- 463
boar 1	TAGG/GGAG	B	65472	75012	TAGG	TAGA	+/+	273	361 +/- 463
boar 1	TAGG/GGAG	B	65472	75014	TAGG	TAGA	+/+	227	361 +/- 463
boar 1	TAGG/GGAG		69974	74999	TAGG	TAGA	+/+	328	361 +/- 463
boar 2	GGAG/gggg		65477	75062	GGAG	TAGA	-/+	220	368 +/- 236
boar 1	GGAG/gggg		65477	75063	GGAG	TAGA	-/+	202	368 +/- 236
boar 1	TAGG/GGAG	A	61043	75084	GGAG	TAGA	-/+	586	368 +/- 236
boar 1	TAGG/GGAG	A	61043	75085	GGAG	TAGA	-/+	198	368 +/- 236
boar 1	TAGG/GGAG	A	61043	75086	GGAG	TAGA	-/+	260	368 +/- 236
boar 1	TAGG/GGAG		65529	75037	GGAG	TAGA	-/+	743	368 +/- 236
boar 2	GGAG/gggg		65973	75108	GGAG	GGAG	-/-	575	
boar 2	GGAG/gggg		65973	75109	GGAG	GGAG	-/-	272	
boar 2	GGAG/gggg		65973	75110	GGAG	GGAG	-/-	299	
boar A	TAGG/GGAG		69974	74998	GGAG	TAGG	-/+	276	
boar 2	GGAG/gggg		65477	75061	GGAG	TAGG	-/+	712	
boar 2	GGAG/gggg		65973	75107	gggg	TAGA	?/+	190	
boar 1	TAGG/GGAG		65529	75036	TAGG	TAGG	+/+	540	
boar 1	TAGG/GGAG	B	65472	75011	TAGG	TGAG	+/?	1410	
boar 1	TAGG/GGAG	B	65472	75010	GGAG	TGAG	-/?	1440	

Haplotypes of the group of markers M11 to M14 were determined on 22 animals from six families; haplotype "gggg" in small letters is a haplotype not previously identified in the 480 LW population; (andro) = expected androstenone level; [CYP11A1] = quantification of *CYP11A1 *expression in the testis

## Conclusion

This study suggests that the variation of *CYP11A1 *expression level is probably not regulated by a mutation located inside the *CYP11A1 *gene but rather by a mutation located downstream of the SNP H3GA0021967. In the French Large White population, the QTL for androstenone is mapped near this SNP. This co-location is probably a coincidence since haplotypes of the M11-M14 region of animals characterised for *CYP11A1 *expression and of animals characterised for the QTL for androstenone are not concordant. This study shows that the testicular expression of *CYP11A1 *is not the main cause of this QTL for androstenone. As a conclusion, we propose to refute the hypothesis that a single mutation located near the centre of the QTL region (M11-M14) could control androstenone accumulation in fat by regulating the CYP11A1 expression.

## Competing interests

The authors declare that they have no competing interests.

## Authors' contributions

AR and KF performed real-time PCR, sequencing, and data processing. AR made the main contributions to the data analysis, data interpretation and drafting of the manuscript. GLM contributed very significantly to data interpretation. CL and JR supervised the experimental design and contributed to data interpretation and manuscript evaluation. Genotyping data acquisition was supervised by CL. All authors read and approved the final manuscript.

## Supplementary Material

Additional file 1 **Presentation of genotypes of animals from the two families evaluated for the CYP11A1 expression**. The data provide genotypes of eight markers allowing the characterisation of SSC7 haplotypes in a large region around *CYP11A1 *of animals from the two main LW families evaluated for *CYP11A1 *expression (families A and B) Click here for file
