# Supplementary material for: New investigations around CYP11A1 and its possible involvement in an androstenone QTL characterised in Large White pigs
Source: Genet Sel Evol. 2011 Apr 19;43(1):15. doi: 10.1186/1297-9686-43-15 (PMC3098772; doi:10.1186/1297-9686-43-15)
Supplement: Additional file 1 — Presentation of genotypes of animals from the two families evaluated for the CYP11A1 expression. The data provide genotypes of eight markers allowing the characterisation of SSC7 haplotypes in a large region around CYP11A1 of animals from the two main LW families evaluated for CYP11A1 expression (families A and B) [file 1297-9686-43-15-S1.PDF]

qPCR on RT products of RNA from testis

Markers	MCS10F15SP6	SW175	SW1418	M11-M14	S0334			MCS10F15SP6	SW175	SW1418	M11-M14	S0334		MCS10F15SP6	SW175	SW1418	M11-M14	S0334
**position on draft 9 in Mb**	42	56.4	64.7	65.91 to 66.11	79.7			42	56.4	64.7	65.91 to 66.11	79.7		42	56.4	64.7	65.91 to 66.11	79.7
							dam						animal					
						family A	61043	9 6	6 2	2 3	TAGA/TAGA	8 7	75082	2 9	6 2	7 3	TAGG/TAGA	6 8
													75083	2 9	6 2	7 3	TAGG/TAGA	6 8
													75084	5 6	5 6	3 2	GGAG/TAGA	2 7
													75085	5 6	5 6	3 2	GGAG/TAGA	2 7
													75086	5 9	5 2	3 3	GGAG/TAGA	2 8
													75087	2 6	6 2	7 3	TAGG/TAGA	6 8
													75088	2 6	6 6	7 2	TAGG/TAGA	6 7
Boar 45881	5 2*	5 6*	7 3*	GGAG/TAGG*	6 2*													
							dam											
						family B	65472	9 3	2 2	3 3	TGAG/TAGA	4 8	75010	5 3	5 2	3 3	GGAG/TGAG	2 4
													75011	2 3	6 2	7 3	TAGG/TGAG	6 4
													75012	2 9	6 2	7 3	TAGG/TAGA	6 8
													75014	2 9	6 2	7 3	TAGG/TAGA	6 8

* deducted genotype

Supplementary table-1: Presentation of genotypes of animals from the two families evaluated for the *CYP11A1* expression

Six litters were produced by inseminating five sows with semen from an LW boar no.45881. In this table we reported only two families of this cross: the A family obtained with dam no.61043 and the B family obtained with dam no.65472.

On the left-hand side of this table we reported genotypes for 4 ordered markers of the SSC7. We were able to deduce that in the A family, animals nos. 75082, 75083 and 75086 had the same maternal SSC7 chromosome whereas animals nos. 75084, 75085 and 75088 had the second maternal SSC7 chromosome. The last descendant of the A family, no.75087, had a maternal recombinant SSC7. In the B family, animals nos. 75010 and 75011 had the same maternal SSC7 whereas animals nos. 75012 and 75014 had the second maternal SSC7.

* deducted genotype
